# Footwear for self-managing knee osteoarthritis symptoms: protocol for the Footstep randomised controlled trial

**DOI:** 10.1186/s12891-018-2144-1

**Published:** 2018-07-18

**Authors:** Kade L. Paterson, Kim L. Bennell, Tim V. Wrigley, Ben R. Metcalf, Penny K. Campbell, Jessica Kazsa, Rana S. Hinman

**Affiliations:** 1 Centre for Health, Exercise and Sports Medicine, Department of Physiotherapy, School of Health Sciences, Faculty of Medicine Dentistry & Health Sciences, The University of Melbourne, Melbourne, VIC Australia; 2 Department of Epidemiology and Preventive Medicine, Monash University, Melbourne, Australia

**Keywords:** Osteoarthritis, Knee, Footwear, Shoes, Clinical trial, RCT, Biomechanics, Clinical trial

## Abstract

**Background:**

Knee osteoarthritis (OA) is a leading cause of musculoskeletal pain and disability globally, and abnormal knee loading is central to disease pathogenesis. Clinical guidelines recommend clinicians provide advice regarding appropriate footwear for people with knee OA, yet there is little research comparing the effects of different footwear on knee OA symptoms. Research suggests that wearing flat flexible shoes is associated with lower knee joint loads compared to stable supportive shoe styles. This two-arm pragmatic, comparative effectiveness randomised controlled trial will compare the effects of daily use of flat flexible shoes and stable supportive shoes on knee OA clinical outcomes, over 6 months.

**Methods:**

164 people with symptomatic medial tibiofemoral OA of moderate to severe radiographic severity (Kellgren and Lawrence Grade 3 & 4) will be recruited from the community. Following baseline assessment, participants will be randomly allocated to receive either i) flat flexible shoes or; ii) stable supportive shoes. Participants will choose two different pairs of shoes from a selection that fulfil the criteria in their allocated shoe class. Limited disclosure will blind participants to group allocation. Participants will be instructed to wear their allocated shoes daily for 6 months (minimum of 6 h/day), after which participants will be reassessed. The primary outcomes are knee pain severity on walking (measured by numerical rating scale) and self-reported physical function (measured by the Western Ontario and McMaster Universities Osteoarthritis Index), assessed at baseline and 6 months. Secondary outcomes include additional measures of knee pain, function, sport and recreation participation and quality-of-life (measured using subscales of the Knee Osteoarthritis Outcome Score), as well as pain at other sites (measured by numerical rating scale), self-reported global ratings of change in pain and physical function (measured by 7-point rating scale), and physical activity levels (measured by Physical Activity Scale for the Elderly).

**Discussion:**

This study will determine whether daily wear of flat flexible shoes improves clinical outcomes in the management of knee OA, compared to stable supportive shoes. Findings will assist clinicians in providing evidence-based advice regarding appropriate footwear for people with knee OA to self-manage symptoms.

**Trial registration:**

Australian New Zealand Clinical Trials Registry reference: ACTRN12617001098325. Registered 28/07/2017.

## Background

Osteoarthritis is the 11th highest contributor to global disability [1]. In 2012, 1.9 million Australians had OA [2], and modelling data estimate a 58% increase in OA by 2032 [2]. The knee joint is commonly affected, and knee OA is extremely debilitating. Pain is dominant, becoming persistent and more limiting as OA progresses. Health expenditure on OA in Australia in 2012 was $3.75 billion AUD [2], with most costs related to conservative and surgical treatments, lost productivity and substantial loss of quality of life. Globally, the annual total direct and indirect cost is estimated to vary between €300 to €19,5000 EUD per patient per year [3].

As OA is a chronic disease with no cure, people with OA have little choice but to self-manage their condition. Accordingly, advice about self-management is the cornerstone of conservative treatment, along with exercise and weight control [4, 5]. As abnormal biomechanics have been implicated in knee OA onset and progression [6, 7], clinical guidelines advocate that clinicians provide advice on “appropriate” footwear to help people with knee OA self-manage their symptoms [4, 8]. However, there is scant evidence from clinical trials to guide footwear choice for this patient group, or indeed, any population with chronic musculoskeletal pain. Guidelines suggest that shoes with thick shock-absorbing soles and arch supports are best for people with OA [8], based largely on expert opinion. Due to the lack of robust clinical trials in this area, footwear trials have been identified as an OA research priority by both the European League Against Rheumatism [8] and the National Institute for Health and Care Excellence (UK) [4].

Abnormal knee joint loading is central to OA pathogenesis [6, 7]. Knee load is typically inferred from three-dimensional gait analysis, usually by parameters of the external peak knee adduction moment. There is some evidence that greater knee load may increase symptoms and risk of structural progression in people with knee OA. For example, an increased knee adduction moment is associated with increased prevalence of medial bone marrow lesions [9] (an important source of pain in knee OA [10]) and medial cartilage defects [11]. The knee adduction moment is also one of the few modifiable factors that predict OA structural progression [7, 12–14]. Reducing knee loads via non-surgical biomechanical treatment strategies is thus an appropriate treatment aim for people with knee OA. Footwear is a promising avenue for self-management, given that foot position and motion influence knee loads.

Biomechanical research shows that wearing shoes significantly increases the knee adduction moment in people with knee OA, compared to barefoot walking [15, 16]. Biomechanical studies also show that some types of footwear increase knee loads more than others [17]. We, and others, have shown that stable supportive shoe styles increase knee loads significantly more compared to flat flexible shoe styles [18–20]. However it is stable supportive shoe styles that are typically recommended by clinicians for people with knee OA, and that are typically worn by people with the condition [21].

There are no high quality clinical trials comparing the effects of flat flexible shoes to stable supportive shoes on knee OA clinical outcomes. Uncontrolled data from the USA show flat flexible shoes reduce knee pain by 36% when worn for 6 h/day over 6 months in 16 people with medial knee OA [22]. In the only randomised controlled trial (RCT) to date [23], a small Brazilian study in 56 women with knee OA compared a standardized flat flexible “Moleca” shoe, worn ≥6 h/day for 6 months, to a control group who wore their own neutral tennis shoes “without characteristics of minimalist footwear”. Flat flexible shoes led to greater pain relief compared to the control group, and significant improvements in function were also observed. Although promising, participants in this study were not blinded, which introduces a high risk of bias given that treatment benefits are often over-estimated when using subjective outcomes with unblinded participants [24]. Thus, further research comparing the effects of flat flexible shoes to stable supportive shoes, using robust RCT designs, is warranted.

The primary aim of this study is to determine if flat flexible shoes lead to significantly greater reductions in knee pain with walking, and improvements in physical function, compared to stable supportive shoes when worn daily over six months. We hypothesise that flat flexible shoes will reduce pain and improve self-reported physical function more than stable supportive shoes.

## Methods/design

### Trial design

This protocol is described according to SPIRIT guidelines for clinical trials ([25]). The trial is a two-arm pragmatic, comparative effectiveness RCT comparing flat flexible shoes to stable supportive shoes. It will be conducted at The University of Melbourne over 3 years. The primary end-point for analysis of outcomes is after 6 months of shoe wear.

### Participants

We will recruit 164 participants with medial tibiofemoral OA from the community via advertisements in print/radio/social media, clinicians and our volunteer database. Knee OA will be classified according to the American College of Rheumatology clinical and radiographic criteria for knee OA [26]. Participants will be included if they:

i) are aged ≥50 years;
ii) report knee pain on most days of the past month;
iii) report average pain during walking (over the previous week) of at least 4 on an 11-point numerical rating scale (NRS, with terminal descriptors of ‘no pain’ and ‘worst pain possible’);
iv) demonstrate moderate-severe (Grade 3–4) tibiofemoral OA on x-ray as determined by the Kellgren & Lawrence grading system [27], as recent research suggests these patients may represent a subgroup to which biomechanical interventions designed to reduce the KAM may be more effectively targeted; and
v) demonstrate tibiofemoral osteophytes on x-ray.

Participants will be excluded if they:

i) have lateral joint space narrowing greater than or equal to medial joint space narrowing on x-ray;
ii) have had knee pain for < 3 months;
iii) have had recent knee surgery (past 6 months) or planning surgery in next 6 months;
iv) currently use shoe orthoses, customized shoes or ankle braces;
v) primarily wear high heels, thongs or work boots that would restrict ability to wear allocated shoes 6 h/day;
vi) have had a hip or knee joint replacement on either side;
vii) have had a high tibial osteotomy on either leg;
viii) have had any knee injections in the past 3 months or planning injections in next 6 months;
ix) self-report any other muscular, joint or neurological condition affecting lower limb function,
x) self-report any systemic or inflammatory joint disease (e.g. rheumatoid arthritis);
xi) currently use, or plan to use, a gait aid in the next 6 months;
xii) do not understand written/spoken English; or
xiii) are unable to commit to study requirements (e.g. wearing shoes, attending appointments, completing outcomes, do not have foot size in the range of 8 to 13US for men, and 6 to 11US for women).

### Procedure

**Fig. 1:**
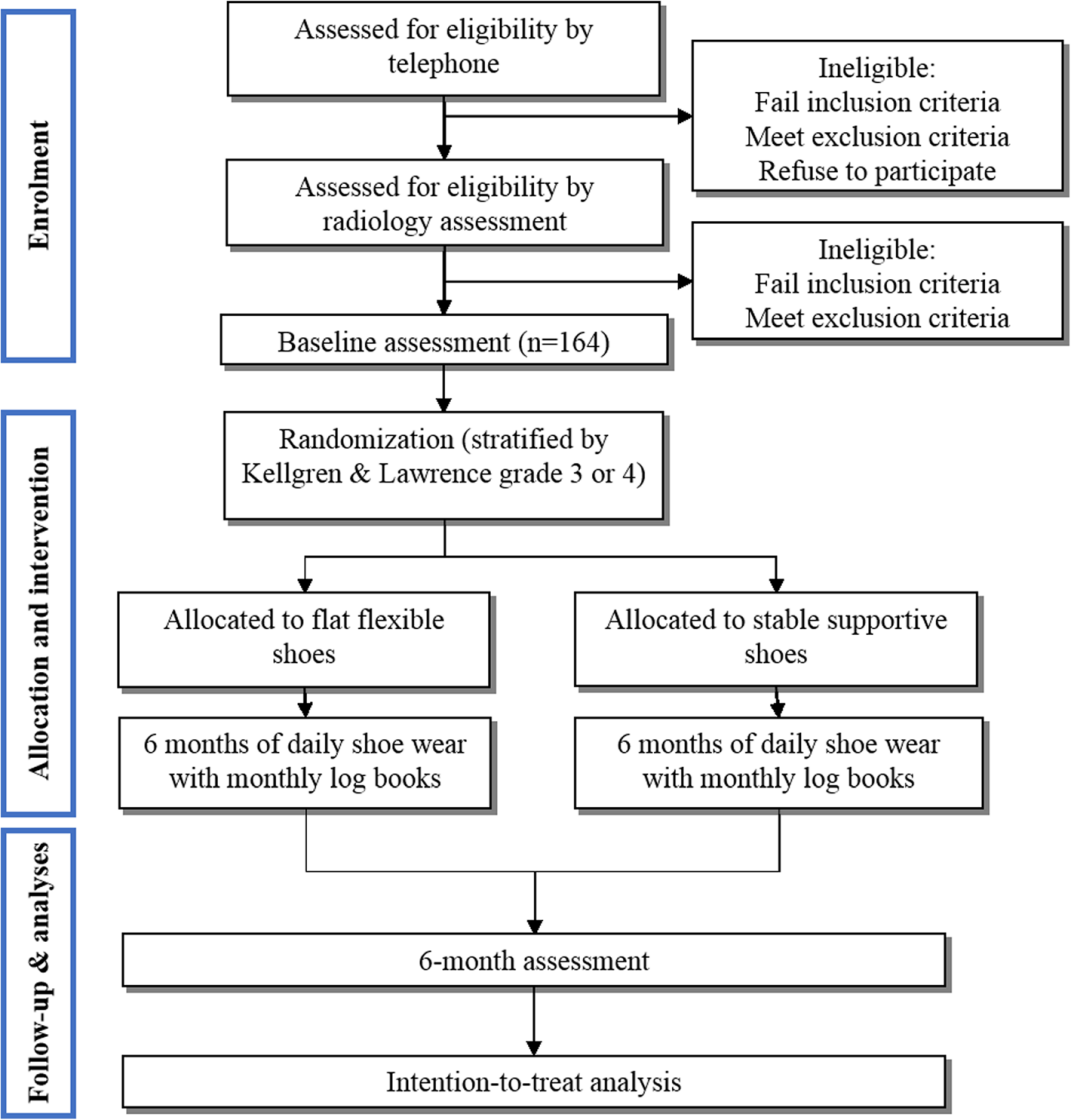
Flow diagram of study phases

Figure 1 outlines the trial phases and Table 1 describes the schedule for enrolment, intervention and assessments. Volunteers will be screened by an online form, then over the telephone by the Trial Coordinator. Potentially eligible participants will undergo standardised posteroanterior standing x-rays. Participants who have undergone standing x-ray in the prior 12 months, and can provide the images to research staff for screening, will not undergo new x-rays due to ethical concerns of exposing them to additional radiation. For participants with bilaterally eligible knees, the most symptomatic knee will be deemed the study knee with respect to outcome measurement.

**Table 1:**
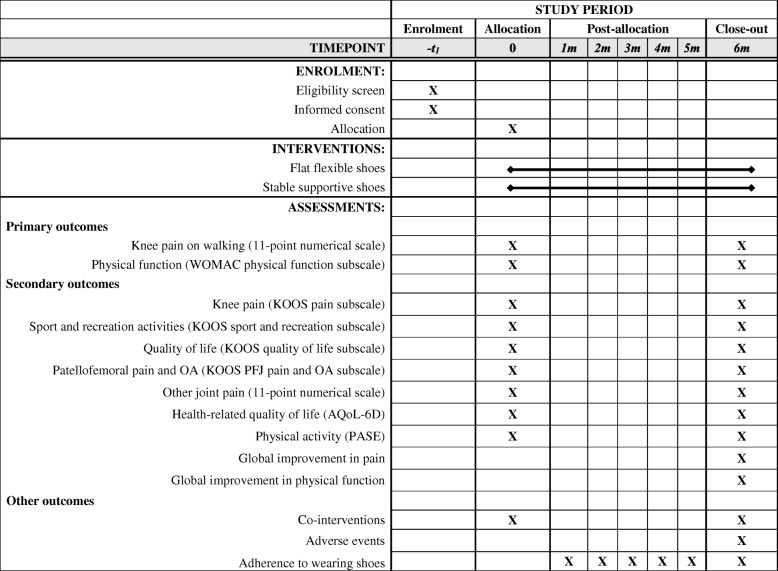
Schedule of enrolment, interventions and assessments

Baseline assessments will be carried out at the Department of Physiotherapy, the University of Melbourne. At the 6-month follow-up, participants will complete questionnaires either on paper or electronically at home. Participants will also complete a short log book to record adherence with allocated footwear for one week of each month during the intervention period. If questionnaires or log books are not returned, the participant will be contacted by email and or phone to prompt their return, or as a last resort, to obtain primary outcome data. Ethical approval has been obtained from the University of Melbourne Human Research Ethics Committee (HREC No. 1748784). All participants will provide written informed consent.

### Randomisation and allocation concealment

Eligible participants will be randomised to receive either flat flexible or stable supportive shoes following baseline data collection of primary and secondary outcomes. Randomisation (generated by the study biostatistician) will be by random permuted blocks (size 6 to 12) and stratified by radiographic disease severity (Kellgren & Lawrence grades 3 and 4 [27]). The schedule will be stored on a password-protected website (REDCap) maintained by a researcher not involved in either participant recruitment or administration of outcome measures. Group allocation will be revealed by this same researcher after baseline outcomes have been completed.

Participants will be blinded to group allocation by the process of limited disclosure. Participants will be informed that the trial is evaluating two different undisclosed “classes” of footwear to compare effects on knee OA symptoms. Participants will not be told about the classes of footwear under investigation, nor the specific shoe make/models that will be evaluated. Participants will not be informed about the study hypotheses, or which group they are allocated to until the study is completed, at which time they will be provided a lay summary of study purpose, hypotheses and findings. As the primary and secondary outcomes are participant-reported, and participants are blinded, this study is also considered assessor-blinded. Research staff administering and entering the participant-reported data will be blinded. Statistical analyses will be performed by a blinded biostatistician. Descriptive baseline characteristics that are researcher-measured using objective methods in the laboratory (e.g. height, weight, foot posture and pressure) will be measured before randomisation by the unblinded staff member who will allocate participants to footwear group, and fit participants to allocated shoes.

### Interventions

We previously identified five criteria (Table 2) that distinguish flat flexible shoes from stable supportive shoes and showed that flat flexible shoes were associated with lower knee joint loads compared to stable supportive shoes [18]. Using our established criteria, we have selected a range of commercially-available shoes for each intervention arm. Once allocated to an intervention group, participants will choose two different pairs of shoes from the range of options in order to maximize adherence.

**Table 2:** Criteria for selecting shoes

	Flat flexible shoes	Stable supportive shoes
Heel height/thickness	< 15 mm	> 30 mm
Shoe pitch	< 10 mm	> 10 mm
Arch support/motion control	Absent	Present
Sole flexibility	“Minimal” rigidity (Footwear Assessment Tool [45])	“Rigid” (Footwear Assessment Tool [45])
Weight^a^	≤200 g	> 300 g

Measurements are based on a size 9 US Men’s and size 9 US Women’s sized shoe

^a^A tolerance of +/− 10% was used for shoe weight

**Fig. 2:**
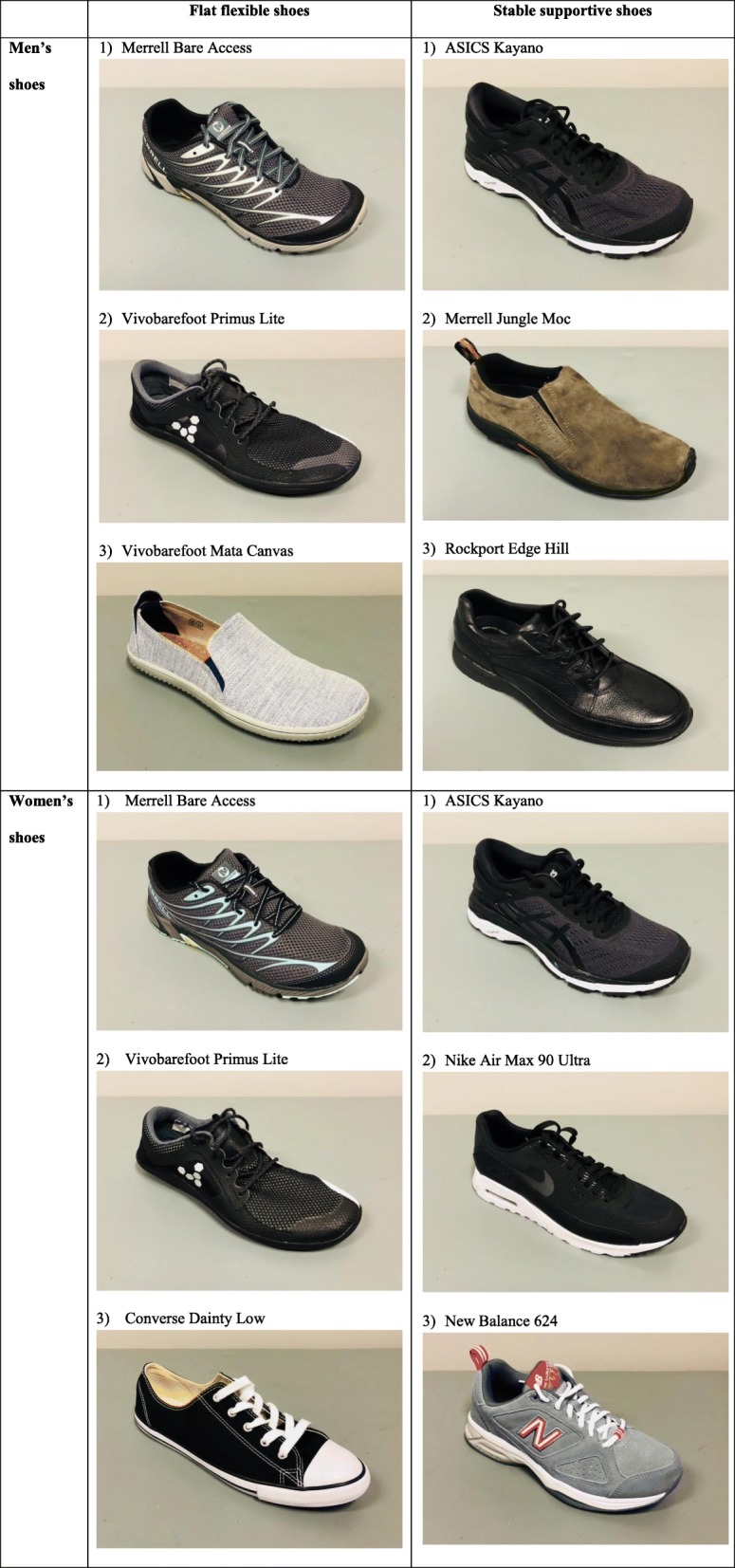
Shoes selected at trial commencement

Consumers with chronic knee pain (68 women and 43 men) were consulted in the trial planning phase to help refine shoe selection for the study to ensure that the chosen shoes would be acceptable and likely to be worn as instructed (minimum of 6 h/day for 6 months). In a survey format, consumers were presented with a wide range of commercially available shoes (that fulfilled the criteria for each shoe class) in different styles and colours. Survey respondents mostly indicated that black was the preferred shoe colour, where black was an option. Where possible, the most popular shoe styles in each class were selected for the trial. These are shown in Fig. 2, and include:

#### Flat flexible shoes

Merrell Bare Access (Men and Women), Vivobarefoot Primus Lite (Men and Women), Vivobarefoot Mata Canvas (Men) and Converse Dainty Low (Women).

#### Stable supportive shoes

ASICS Kayano (Men and Women), Merrell Jungle Moc (Men), Nike Air Max 90 Ultra (Women), Rockport Edge Hill (Men) and New Balance 624 (Women).

As footwear manufacturers frequently change shoe models from season to season and year to year, we expect that shoes readily available at trial commencement may cease to be available during the course of the trial or may be rebranded with a different shoe name. In such instances, shoes will be replaced by another pair of commercially available shoes that fulfil the listed criteria in Table 2.

Participants will be instructed to wear either pair of their allocated shoes as much as possible every day for 6 months, and to avoid wearing their own usual footwear as much as possible. At a minimum, participants will be asked to wear the shoes for at least 6 h every day.

### Outcome measures

Table 1 summarises the outcome measures that are being collected for this study. Our primary outcomes are validated measures of pain and physical function that have been recommended for use in knee OA clinical trials [28]. Conclusions regarding treatment efficacy will be based on the 6-month changes in our primary outcome measures. Our two primary outcomes, measured at baseline and 6 months, are:

- *Severity of knee pain during walking, scored on an 11-point NRS*. Average overall pain on walking in the last week will be self-reported using an 11-point NRS. Scores range from 0 to 10, where 0 = no pain and 10 = worst pain possible. This outcome measure has demonstrated reliability in OA [29].
- *Physical function subscale of the WOMAC.* Limitations with physical functioning will be measured by the Western Ontario and McMaster Universities (WOMAC) Osteoarthritis Index (Likert version 3.1) [30]. The WOMAC is a self-report, disease-specific instrument which has established validity, reliability and responsiveness in an extensive range of OA studies [31]. The WOMAC physical function subscale contains 17 questions regarding knee function over the past week, with Likert response options ranging from 0 (no dysfunction) to 4 (extreme dysfunction). WOMAC scores will be extracted from the Knee Injury and Osteoarthritis Outcome Score (KOOS) [32] questionnaire, which contains the WOMAC questions. Total score ranges from 0 to 68, with higher scores indicating worse function.

Secondary outcome measures will be administered at baseline and at 6 months unless otherwise indicated. These include:

- *Pain subscale of the KOOS*. The subscale is scored using 9 questions about knee pain experienced in the last week, with Likert response options from None to Extreme. Scores range from 0 to 100, with lower scores indicating worse pain [32].
- *Sport and recreation subscale of the KOOS.* The subscale is scored using five questions regarding function with sport and recreational activities in the last week, with Likert response options ranging from None to Extreme [32]. Scores range from 0 to 100, with lower scores indicating worse function [32].
- *Quality of life subscale of the KOOS*. The subscale consists of four questions about knee-related quality of life experienced in the last week, with five Likert response options for each question. Scores range from 0 to 100, with lower scores indicating worse quality of life [32].
- *Patellofemoral pain and OA subscale of the KOOS.* The subscale is comprised of 11 questions about knee pain and function experienced in the last week, with five Likert response options for each question. Scores range from 0 to 100, with lower scores indicating worse patellofemoral symptoms [33].
- *Lower limb pain severity*. Average overall pain in the last week will be recorded using an 11-point NRS (0 = no pain and 10 = worst pain possible) at each of the following sites: (i) study knee, (ii) contralateral knee, (iii) ipsilateral hip, (iv) contralateral hip, (v) ipsilateral foot/ankle, (vi) contralateral foot/ankle, and (vii) back.
- *Participant-perceived global change.* At 6 months, participants will rate their overall global change in a) pain and b) physical function since enrolling in the study. Response to treatment will be scored using a 7-point global rating of change Likert scale, with terminal descriptors of ‘much worse’ to ‘much better’ [34]. Participants who report ‘moderately better’ or ‘much better’ will be classified as improved. All other respondents will be classified as not improved.
- *Health-related quality of life.* The Assessment of Quality of Life (AQoL) [35] (version AQoL-6D) will be used to measure health-related quality of life. It consists of 20 items that assess independent living, mental health, relationships, pain, coping and senses. Scores range from − 0.04 to 1.00 with higher scores indicating better quality of life.
- *Physical activity levels.* The Physical Activity Scale for the Elderly (PASE) will be used to measure physical activity over the previous week [36]. Scores range from 0 to over 400, with higher scores indicating greater physical activity.

### Treatment adherence

Participants will rate their perceived overall level of adherence over the past 6 months with the instruction to wear their allocated shoes for a minimum of 6 h per day on an 11-point NRS (with terminal descriptors of ‘shoes not worn all’ and ‘shoes worn completely as instructed’). At the 6-month follow up assessment, participants will also indicate whether they stopped wearing either pair of study shoes during the 6 months on a categorical scale (Yes or No). Participants who score Yes will describe when and why they ceased wearing their allocated shoes, and this will be reported descriptively. Finally, participants will record in log books how many hours each day they wear their allocated shoes, for 7 consecutive days every month, over the 6-month intervention period.

### Adverse effects of treatment and co-interventions

Adverse events will be defined as any problem experienced in the study knee or elsewhere in the body because of wearing the study shoes. Adverse events will be self-reported by participants using a custom-developed table, and by open-ended questioning at 6 months. Participants will also be asked to contact the researchers at any time by telephone or email to report adverse events. Use of co-interventions (medications for knee pain and any other treatments for knee OA) will also be recorded at baseline and 6-months. Participants will complete a custom-developed table to indicate the frequency of use (over the past 6 months) of a range of pain and arthritis medications and co-interventions.

### Descriptive measures

A range of other measures will be collected to describe demographic characteristics, treatment expectations, self-efficacy, foot biomechanics and current footwear characteristics. These measures will not be used to determine treatment efficacy but will be used for descriptive purposes. They include height, weight and body mass index; radiographic disease severity using the Kellgren & Lawrence scale [27]; current employment status; a rating of participant expectation of treatment outcome, measured on a 5-point ordinal scale; self-efficacy, determined using the Arthritis Self Efficacy Scale [37]; objective measures of foot posture, mobility and function including the Foot Posture Index (FPI [38]), Foot Mobility Magnitude [39], navicular drop [40] and in-shoe regional foot pressure patterns (Novel Pedar, Munich, Germany) during walking; and characteristics of each participant’s three most commonly worn shoes, including shoe weight, heel height, pitch, motion control properties and flexibility [18].

### Sample size calculations

We aim to detect the minimal clinically important difference (MCID) on primary outcomes between groups (1.8 (out of 10) [41] for NRS pain and 6 (out of 68) for WOMAC function) [42]. We assume between-participant standard deviations of 2.7 and 11.4, and baseline to 6-month correlations of 0.21 and 0.39 for pain and function respectively (data from our footwear trial in a similar sample) [43]. Using analysis of covariance adjusted for baseline score, we need 46 per arm to achieve 90% power to detect MCID in pain and 65 per arm for function. Allowing for 20% attrition, we will recruit 82 people per arm (*n* = 164).

### Statistical analyses

A biostatistician will analyse data in a blinded manner, with *p* values less than 0.05 considered significant. Main comparative analyses between groups will be performed using intention-to-treat. If more than 5% of primary outcomes are missing, multiple imputation will be applied. For the primary hypothesis, differences in mean change in pain and function (baseline minus follow-up) will be compared between groups using linear regression modelling adjusted for baseline values and the stratifying variable of Kellgren & Lawrence grade. Similar analyses will be conducted for continuous secondary outcomes. Improvement based on global change will be compared across groups using risk differences, calculated from fitted logistic regression models. The effect of patient characteristics on outcomes will be explored by including relevant terms as covariates in models. Standard diagnostic plots will be used to check model assumptions.

To assess whether the effect of shoe class on the primary outcomes is moderated by any of Kellgren & Lawrence grade, Foot Posture Index score, body mass index or baseline score on the KOOS patellofemoral pain and OA subscale, appropriate interaction terms between randomised group and each of these variables will be included in regression models for the primary outcomes, and for each potential effect modifier separately. For the continuous moderators, a fractional polynomial approach will be applied [44].

### Timelines

The application for project funding was successful in October 2016 and funding commenced in August 2017. Ethics approval was obtained from the Human Research Ethics Committee of the University of Melbourne in May 2017. Recruitment commenced in August 2017 and will be completed in December 2019. The trial is due for completion in July 2020 when all participants will have completed 6-month follow-up.

## Discussion

This paper has outlined the theoretical foundation, and presented the protocol, for a two-arm pragmatic, comparative effectiveness RCT comparing flat flexible to stable supportive shoes for people with knee OA. Clinical OA guidelines recommend appropriate footwear for knee OA self-management [4, 8], with some guidelines specifying shoes with supportive features, based solely on expert opinion [8]. Stable supportive footwear are also recommended by clinicians for people with knee OA, and are typically worn by people with the disease [21]. Yet these styles increase knee loads compared to flat flexible shoes [18–20], while limited clinical data suggests flat flexible shoes may lead to symptomatic benefits [23]. Given the lack of high quality RCTs comparing footwear styles, leading international organisations [4, 8] have highlighted footwear clinical trials as an important OA research priority. Findings from the Footstep trial will assist clinicians and patients with knee OA in selecting the most appropriate footwear to help self-manage symptoms associated with the disease.

## Abbreviations

AQoL: Assessment of Quality of Life
CONSORT: Consolidated Standards of Reporting Trials
HREC: Human Research Ethics Committee
KOOS: Knee Injury and Osteoarthritis Outcome Score
MCID: Minimal clinically important difference
NRS: Numerical rating scale
OA: Osteoarthritis
PASE: Physical Activity Scale for the Elderly
RCT: Randomised controlled trial
WOMAC: Western Ontario and McMaster Universities
